# Enterovirus D68 receptor usage: from static attachment to dynamic entry

**DOI:** 10.1128/jvi.01949-25

**Published:** 2025-12-30

**Authors:** Dongxue Liu, Zhilin Ji, Xiangyu Zheng, Huiming Xia, Wanshan Yang, Peng-Fei Ge, Wei Wei

**Affiliations:** 1 Institute of Virology and AIDS Research, The First Hospital of Jilin University 117971, Changchun, Jilin, China; 2 Department of Pathology, Medical College, Yanbian University 12396, Yanji, Jilin, China; 3 Department of Neurosurgery, The First Hospital of Jilin University 117971, Changchun, Jilin, China; 4 Department of Neurology and Neuroscience Center, The First Hospital of Jilin University 117971, Changchun, Jilin, China; 5 Cancer Center, Tumor Research Institute of Jilin University, Key Laboratory of Organ Regeneration and Transplantation of Ministry of Education, The First Hospital of Jilin University 117971, Changchun, Jilin, China; Universiteit Gent, Merelbeke, Belgium

**Keywords:** enterovirus D68, viral receptor, sialic acid, ICAM-5, MFSD6, acute flaccid myelitis, viral entry

## Abstract

Enterovirus D68 (EV-D68) is a globally reemerging respiratory pathogen of notable clinical concern due to its association with severe respiratory disease and the paralytic complication acute flaccid myelitis (AFM). Viral tropism and pathogenesis are critically dictated by interactions with host cell receptors. Our understanding of this process has evolved from a simple model of sialic acid dependence to a dynamic paradigm involving a repertoire of attachment factors and proteinaceous entry receptors. This review synthesizes the evolving landscape of EV-D68 receptor usage. We detail the well-established role of α2,6-linked sialic acid as an attachment factor and uncoating trigger for historical strains. We further discuss the discovery of intracellular adhesion molecule-5 (ICAM-5) as a neuron-specific receptor that provides a molecular explanation for neurotropism in AFM. A pivotal recent advance is the identification of major facilitator superfamily domain-containing 6 (MFSD6) as an essential entry receptor for a broad range of EV-D68 strains in both respiratory and neuronal cells. We explore the implications of this receptor versatility, whereby the virus can switch between or co-opt sialic acid, ICAM-5, and MFSD6, a plasticity that influences tissue tropism and viral evolution. Finally, we highlight how these mechanistic insights, particularly the characterization of the MFSD6 interface, are paving the way for novel therapeutic strategies, such as engineered decoy receptors, and outline key future directions in the field.

## INTRODUCTION

Enteroviruses constitute a large genus of positive-sense, single-stranded RNA viruses within the family *Picornaviridae*. Historically classified into serogroups, such as polioviruses, coxsackieviruses, echoviruses, and numbered enteroviruses, they are now taxonomically organized into four species: *Enterovirus A* to *Enterovirus D*. The nonenveloped virion exhibits icosahedral symmetry and encapsulates an approximately 7.5 kb RNA genome, which is translated into a single polyprotein. Proteolytic processing yields both structural (VP1–VP4) and nonstructural proteins (1–4). Recent studies have revealed an additional open reading frame (ORF2) in the enterovirus genome, encoding a protein (ORF2p/uORF) that facilitates viral replication in human intestinal epithelial cells (5, 6).

**Fig 1:**
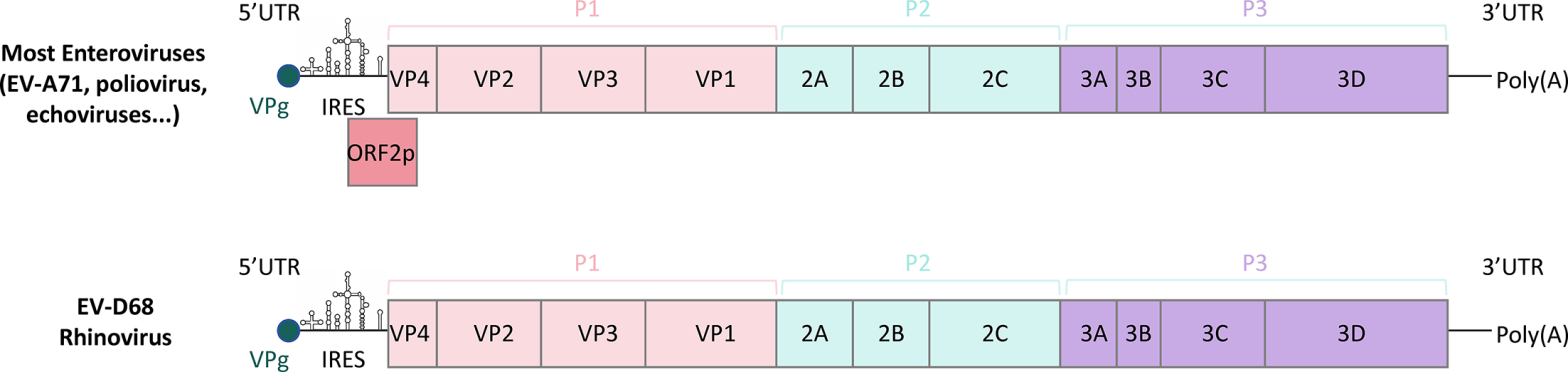
Comparison of the genomic structure of EV-D68 with that of other enteroviruses. UTR, untranslated region. IRES, internal ribosome entry site.

Enterovirus D68 (EV-D68), a member of the species *Enterovirus D*, is distinguished from most enteroviruses by its primary tropism for the respiratory tract rather than the gastrointestinal system (7). It causes a spectrum of respiratory illnesses, ranging from mild cold-like symptoms to severe bronchiolitis and pneumonia (8). Unlike other enteroviruses, which are acid-resistant and replicate in the human gastrointestinal tract, EV-D68 is acid-labile, prefers a lower growth temperature, and replicates primarily in the respiratory tract; these traits are characteristic of human rhinoviruses (9–11). Moreover, the EV-D68 genome lacks the ability to encode ORF2p, which may further explain its inability to efficiently propagate via the fecal-oral route, which is a hallmark of most classic enteroviruses (Fig. 1) (5). Since 2014, global outbreaks of EV-D68 have been strongly associated with an increase in cases of acute flaccid myelitis (AFM), a poliomyelitis-like neurologic disorder, underscoring its growing public health threat (10, 12, 13).

**Fig 2:**
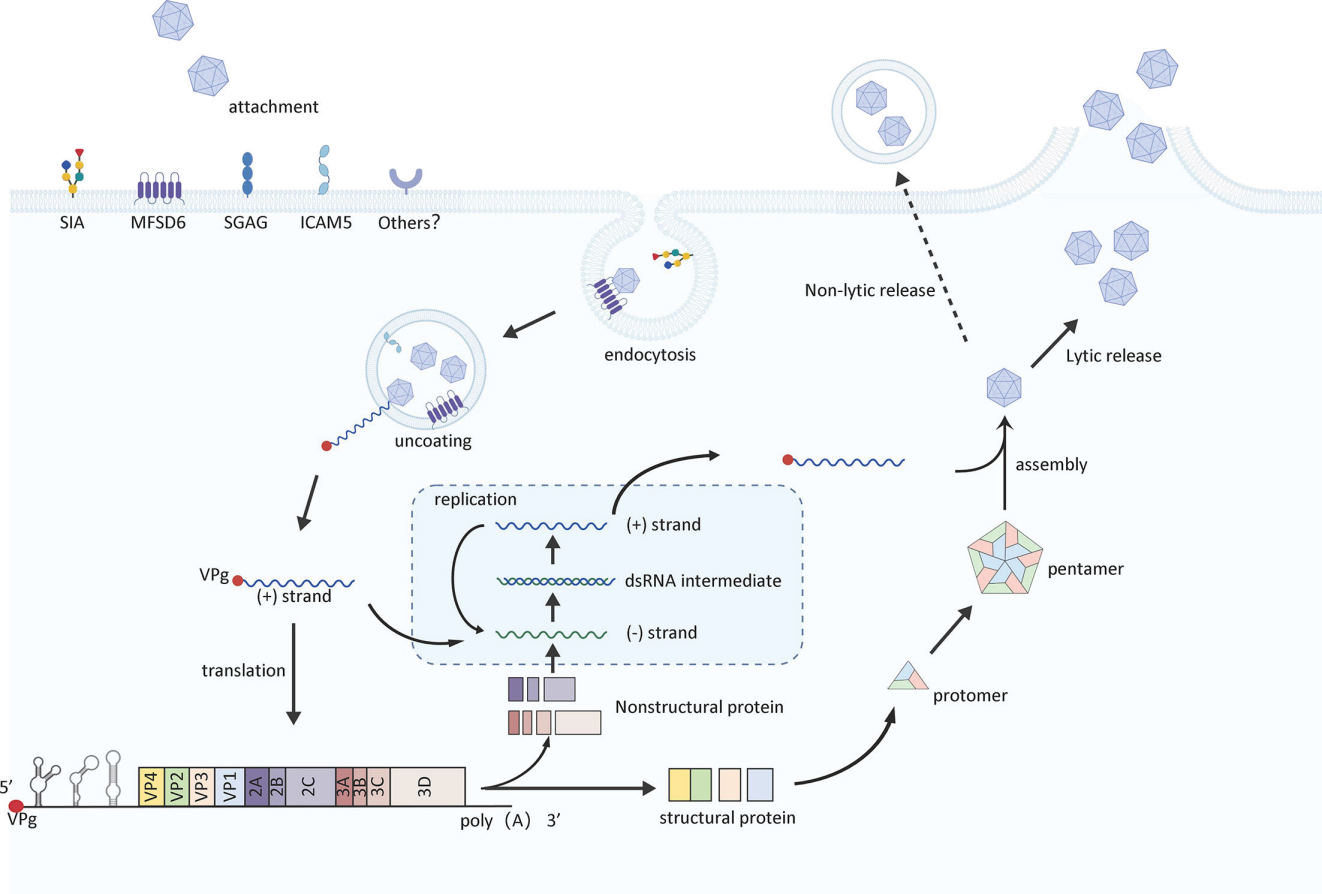
Receptor-mediated EV-D68 infection and replication.

The life cycle of enteroviruses begins with attachment to specific cell surface receptors, followed by receptor-mediated endocytosis, uncoating, and the release of genomic RNA into the cytoplasm for translation and replication (14). Progeny virions are assembled and released upon cell lysis (14). This initial receptor interaction is a key determinant of tropism, host range, and pathogenesis (15, 16). With respect to EV-D68, our understanding of receptor usage has evolved considerably (Fig. 2). Early studies identified sialic acid as an important attachment factor (17); however, sialic acid alone could not explain the neurotropism and heightened virulence of contemporary strains. The subsequent identification of protein receptors, such as intracellular adhesion molecule-5 (ICAM-5) (18), offered a plausible explanation for how EV-D68 could directly infect the neural tissue, providing key evidence supporting a neurotropic pathway. A major recent breakthrough is the discovery by us and others of major facilitator superfamily domain-containing 6 (MFSD6) as a functional entry receptor for EV-D68 across diverse cell types, including respiratory and neuronal cells (19, 20). These findings not only address key questions about viral entry but also have accelerated the development of novel antiviral strategies (Table 1).

**TABLE 1:** The function and characteristics of the EV-D68 receptors

	Sialic acid	ICAM-5	MFSD6
Receptor function	Attachment factor	Attachment factor, uncoating receptor	Attachment factor, uncoating receptor
Strain fependence	Yes	Not observed	Not observed
Tissue distribution	Widely distributed	Predominantly expressed in neuronal tissues, particularly in the telencephalon	Expressed in multiple tissues, including lung, gastrointestinal tract, blood, and nervous tissue
Limitations	Some EV-D68 epidemic strains can infect without sialic acid	Limited expression profile of ICAM-5	MFSD6 depletion does not entirely prevent EV-D68 infection
Targeted therapeutic strategies	DAS181	Soluble ICAM-5-Fc fragments	Engineered decoy receptor FSD6-Fc(CH3)

This review synthesizes current knowledge on EV-D68 receptor usage, tracing the evolution from sialic acid dependence to the characterization of specific protein receptors and cofactors, with emphasis on the pivotal role of MFSD6. We also discuss how these interactions shape viral evolution and disease outcomes.

## SIALIC ACID: THE HISTORICAL ATTACHMENT RECEPTOR

Sialic acid (Sia) was one of the earliest identified receptors for EV-D68, and its role in viral entry has been extensively studied, particularly for historical prototype strains (e.g., Fermon), which show high dependence (17, 21–23). The EV-D68 capsid features a conserved “canyon” structure, a receptor-binding site common to many picornaviruses that provides protection from immune surveillance (17, 24). Structural biology studies have revealed that the canyon floor can bind sialylated glycans, inducing conformational changes in the viral particle that initiate the uncoating process.

### Specificity and structural basis of sialic acid binding

Liu et al. were the first to elucidate the binding mode between EV-D68 and sialylated glycans, such as the trisaccharide 6′-sialyllactosamine (6′SLN), via X-ray crystallography (17). They identified the binding site at the “east end” of the canyon formed by contributions from both VP1 and VP3. The sialic acid moiety engages with conserved amino acids (e.g., Arg1270 and Asp3232) via hydrogen bonds and electrostatic interactions involving its carboxylate and N-acetyl groups. This binding mode differs from those of immunoglobulin-like receptors, which typically interact directly with the GH loop of VP1.

Critically, sialic acid binding induces a series of long-range conformational changes in the viral capsid (17). Notably, it triggers the movement of the VP1 GH loop toward the hydrophobic pocket, leading to the expulsion of the “pocket factor” (a fatty acid-like molecule). This event destabilizes the virion, priming it for subsequent uncoating. This mechanism is functionally conserved, with the uncoating triggered by immunoglobulin-like receptors, suggesting that the canyon serves as a universal platform for the virus to “sense” receptor engagement.

### Sialic acid linkage and tissue tropism

EV-D68 exhibits a marked preference for α2,6-linked sialic acid over the α2,3-linked form, a specificity first systematically demonstrated for both historical and contemporary strains using glycan array and hemagglutination assays (23). Because α2,6-linked sialic acids are predominantly expressed on the surface of human upper respiratory tract epithelial cells, this specificity aligns well with the respiratory tropism of the virus. Using CRISPR-Cas9 knockout and glycosyltransferase-mutant cell lines, Baggen et al. (22) further confirmed that although EV-D68 can utilize both linkage types for infection, α2,6-linked sialic acid is significantly more efficient (22). This finding broadens our understanding of the tissue tropism of the virus, suggesting the potential for infection of the lower respiratory tract as well.

### Strain-dependent variation in sialic acid reliance

Although sialic acid is crucial for early strains, growing evidence indicates that many contemporary circulating strains have evolved sialic acid-independent infection pathways. Through haploid genetic screens, Baggen et al. (22) demonstrated that certain clinical isolates could replicate efficiently in cells lacking sialic acid, suggesting the ability to use nonsialic acid receptors (22). Interestingly, these strains often retain the ability to bind sialic acid (e.g., hemagglutination-positive); however, their infectivity does not depend on this binding, suggesting that for these strains, sialic acid may function merely as an attachment factor or coreceptor rather than as an essential entry receptor.

### Synergistic roles of sialic acid and other glycans

Recent research has expanded the spectrum of glycans recognized by EV-D68. Utilizing the glycan array technology, Pereirinha da Silva et al. (25) reported that some EV-D68 strains can bind to not only α2,6-linked N-glycans but also α2,8-linked sialic acid structures found in gangliosides (e.g., GD3, GT1a, and GQ1b), indicating the ability to recognize glycolipid receptors (25). Inhibitory experiments using glycolipid synthesis inhibitors and glycomimetic compounds confirmed that these gangliosides can function as functional receptors that mediate viral entry likely by promoting the conformational changes required for uncoating.

Furthermore, some strains have acquired mutations, such as an E→K/R substitution at position 271 of VP1, which confers the ability to bind heparan sulfate (HS) (26). This glycosaminoglycan is abundant on cell surfaces and in the extracellular matrix, and its binding may increase viral attachment efficiency in specific cell types. However, studies by Sridhar et al. (27) using human airway organoids and brain organoids revealed that HS binding did not significantly enhance neurotropism (27), suggesting that it might be a cell culture adaptation whose physiological relevance requires further validation. One plausible explanation for the emergence of such variants is that EV-D68 faces diverse selective pressures during infection and transmission, favoring phenotypic plasticity in receptor usage. Under specific conditions, the E271K/R mutation or analogous changes that strengthen HS affinity may shift the virus’ entry preference toward HS-mediated uptake, thereby circumventing host-imposed bottlenecks. Indeed, emerging evidence indicates that sulfated glycosaminoglycan-binding EV-D68 strains can bypass PLA2G16, a pan-enterovirus host factor essential for productive uncoating (26). While the *in vivo* significance of this alternative entry route remains to be fully established, the capacity to exploit heparan sulfate proteoglycans as a backup entry pathway underscores the remarkable adaptability of EV-D68 in navigating heterogeneous host receptor landscapes.

In summary, the role of sialic acid in EV-D68 entry is multifaceted and strain-specific. Although it demonstrates clear structural specificity by preferentially binding α2,6-linked residues within the canyon floor, its functional importance spans a spectrum from being an essential trigger for uncoating in historical strains to serving primarily as an attachment factor or even being dispensable for many contemporary, sialic acid-independent strains (22). Furthermore, sialic acid does not act in isolation; it can function synergistically with other glycans, such as gangliosides and heparan sulfate, to broaden the viral receptor repertoire (26). The physiological relevance of these interactions is also context-dependent, as sialic acid remains a primary receptor in human airway models but appears to play a more limited role in neuronal tissues, underscoring the complex and evolving relationship between EV-D68 and its glycan receptors.

## PROTEIN RECEPTORS: UNVEILING THE ENTRY MECHANISMS

### ICAM-5/Telencephalin: a neuronal receptor for EV-D68 entry

The identification of intercellular adhesion molecule-5 (ICAM-5) as a functional receptor for EV-D68 marked a pivotal breakthrough in understanding the neurotropic potential of the virus and its association with acute flaccid myelitis.

ICAM-5, also known as telencephalin, is a neuron-specific intercellular adhesion molecule predominantly expressed in the telencephalon, including the cerebral cortex and the hippocampus, and is expressed at a lower level in human respiratory tissues (28–31). Its expression is largely confined to dendrites and neuronal cell bodies, providing a molecular basis for the potential of EV-D68 to infect neural tissues, particularly during severe infections when the virus may cross the blood-brain barrier.

The role of ICAM-5 as a bona fide entry receptor for EV-D68 has been rigorously validated through multiple lines of evidence. EV-D68 virions bind specifically and efficiently to soluble ICAM-5-Fc proteins *in vitro*, as shown by virion capture assays (18, 32, 33). Functionally, silencing ICAM-5 expression in permissive cells (e.g., HEK293T and A172 cells) significantly attenuated EV-D68 replication and cytopathic effects. Conversely, the ectopic expression of ICAM-5 in nonpermissive Vero cells increased the susceptibility to EV-D68 infection, supporting viral RNA replication, virion production, and CPE induction. The critical role of ICAM-5 was further underscored by the potent inhibition of EV-D68 infection using soluble ICAM-5 fragments in physiologically relevant models, including primary human bronchial epithelial cells, rat hippocampal neurons, and mouse brain tissues, highlighting its functional importance across diverse species and cell types.

These findings have been strongly complemented and extended by independent genetic studies. Elegant work in a near-haploid human HAP1 cell system confirmed that both genetic knockout of ICAM-5 and treatment with soluble ICAM-5 efficiently inhibited infection by contemporary strains, such as EV-D68-947 (26). Notably, this inhibition occurred independently of sialic acid, clearly establishing ICAM-5 as a functional protein receptor whose role is distinct from and can operate in parallel to the glycan-mediated attachment mechanism (18).

Cryo-electron microscopy (cryo-EM) revealed that ICAM-5 binding induces the conversion of mature EV-D68 virions into A-particles—an expanded conformational state that promotes genome release (32). This transformation mirrors the uncoating mechanisms characterized in other picornaviruses (34–37), underscoring the role of ICAM-5 not only in viral attachment but also in triggering downstream entry processes.

Recent evidence suggests that ICAM-5 is recruited to lipid rafts during EV-D68 infection, which are cholesterol-rich membrane microdomains involved in viral entry and signaling. Methyl-β-cyclodextrin (MβCD), a lipid raft-disrupting agent, was shown to inhibit EV-D68 infection by preventing the colocalization of ICAM-5 and viral particles in these membrane domains (38). These findings suggest that ICAM-5 function may be spatially regulated within the plasma membrane and that lipid rafts serve as platforms for efficient receptor-mediated entry.

Although ICAM-5 can function independently of sialic acid, many EV-D68 strains (e.g., Fermon) still require sialic acid for efficient infection, even in ICAM-5-expressing cells. This dual dependency suggests a model in which ICAM-5 acts as a protein receptor that may cooperatively enhance sialic acid-mediated viral entry or stabilize virus–receptor interactions. Recent studies have indicated that certain circulating strains (e.g., EV-D68-947) can utilize sulfated glycosaminoglycans (sGAGs) as alternative receptors (26).

Despite strong evidence supporting the role of ICAM-5 in EV-D68 infection, its restricted expression pattern does not fully account for the efficient replication of the virus (20, 39), suggesting the presence of additional ubiquitously expressed receptors or coreceptors. Furthermore, evidence supporting ICAM-5-dependent viral internalization in host cells is lacking. Moreover, the structural details of ICAM-5-capsid interactions remain poorly understood, and the potential for receptor usage plasticity in emerging strains underscores the need for ongoing surveillance.

### Major facilitator superfamily domain-contaia key entry receptorning 6 (MFSD6): a key entry receptor

Recent identification of major facilitator superfamily domain-containing 6 (MFSD6) as an entry receptor for EV-D68 represents a transformative advance, explaining the efficient replication of the virus in the respiratory epithelium and its broad tissue tropism (19, 20). Three concurrent studies have unequivocally established the critical role of MFSD6 through functional genomics, structural biology, and therapeutic validation.

The discovery of MFSD6 emerged from independent, genome-wide loss-of-function screens. RNA interference (RNAi) screening of membrane proteins in EV-D68-susceptible HeLa cells revealed that MFSD6 was the most significant hit (19). In parallel, Varanese et al. (20) performed CRISPR-Cas9 knockout screens in human lung (A549) and glioblastoma (U87-MG) cells, revealing the strong enrichment of guides targeting MFSD6 in cells resistant to EV-D68 infection (20). Subsequent validation across multiple cell lines, including primary human bronchial epithelial cells (HBECs) and neuronal models, confirmed that ablation of MFSD6 expression drastically inhibited viral RNA replication, protein synthesis, and the production of infectious progeny. This requirement was conserved across historical (Fermon) and contemporary circulating strains (e.g., US/MO/14-18947), highlighting MFSD6 as a nonredundant, broad-spectrum entry factor.

**Fig 3:**
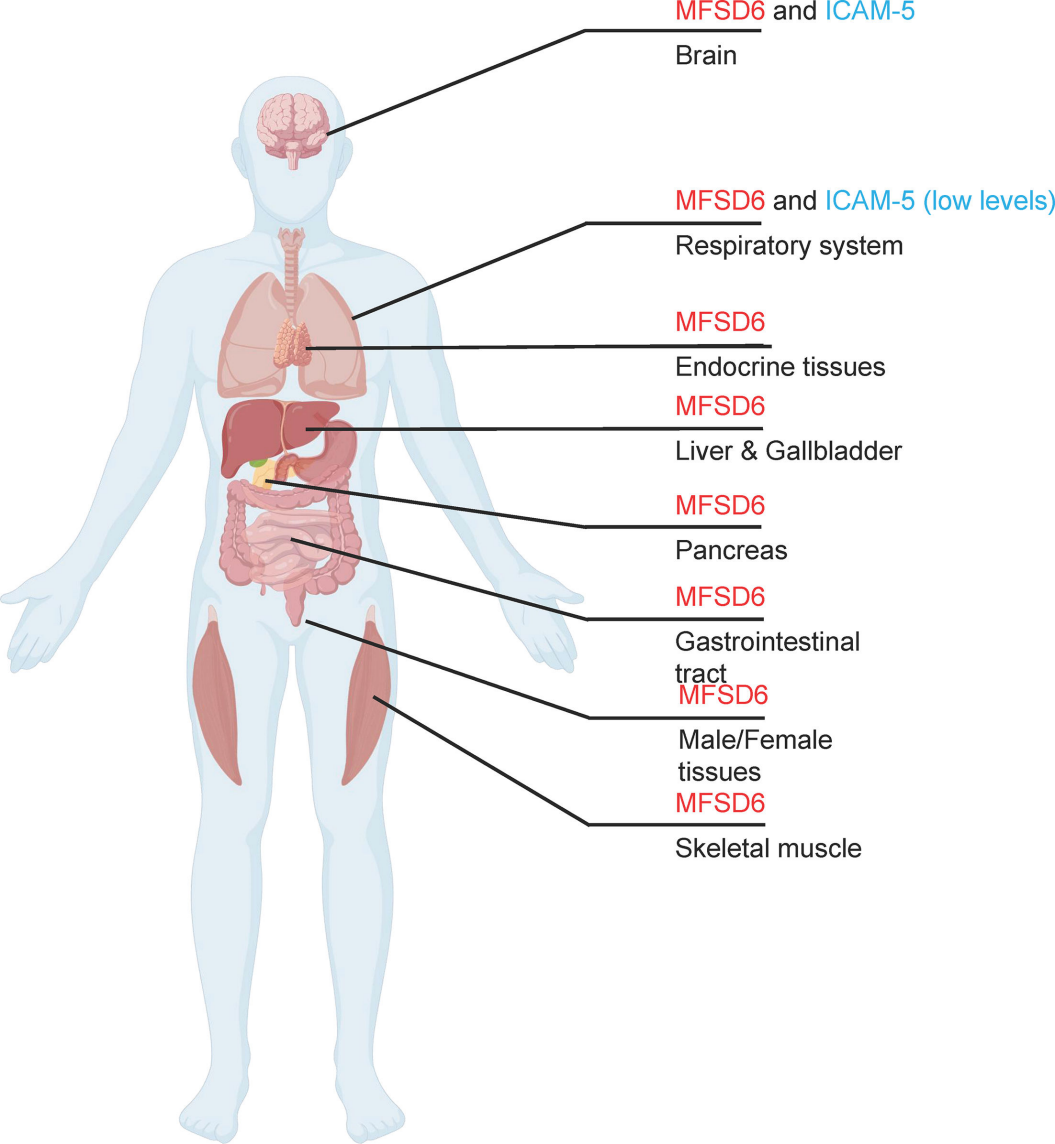
Tissue expression profiles of EV-D68 protein receptors, ICAM-5 and MFSD6. The data are referenced from The Human Protein Atlas Project (https://www.proteinatlas.org/).

In contrast to the neuron-restricted expression of ICAM-5, MFSD6 is widely expressed across human tissues (Fig. 3). Database analyses and experimental data indicate significant MFSD6 mRNA and protein levels in tissues relevant to EV-D68 pathogenesis, including the respiratory tract (e.g., nasal epithelium and lungs), central nervous system, and gastrointestinal tract (https://www.proteinatlas.org/). This ubiquitous expression pattern aligns with the primary respiratory transmission route of EV-D68 and its potential for systemic spread, including neuroinvasion. The broad presence of MFSD6 provides a molecular explanation for the ability of the virus to efficiently infect airway cells, a tropism not fully accounted for by previously identified receptors.

MFSD6 has been rigorously validated as a bona fide entry receptor. Loss-of-function studies demonstrated that MFSD6 deficiency specifically impaired viral internalization with a minimal effect on initial, sialic acid-dependent attachment, highlighting its role in postattachment entry steps. Crucially, gain-of-function experiments revealed that ectopic expression of human MFSD6 in nonpermissive cells (e.g., Vero cells and MDCK cells) renders them highly susceptible to EV-D68 infection, supporting full viral replication cycles. Biochemical assays confirmed a direct, specific interaction between MFSD6 and EV-D68 virions. Furthermore, MFSD6 colocalizes with viral particles at the plasma membrane during early infection, providing spatial evidence for its receptor function. However, MFSD6 is unlikely to be the sole mediator of viral entry. The approximately 10-fold reduction in viral infectivity following MFSD6 knockout, as demonstrated by both Liu et al. and Varanese et al., still leaves a significant level of residual infectivity that cannot be disregarded, implying the existence of additional yet-to-be-identified receptors or host factors capable of facilitating MFSD6-independent viral entry.

The molecular details of the MFSD6-EV-D68 interaction were elucidated by high-resolution cryo-electron microscopy of the structure of the virus bound to a soluble MFSD6 fragment. The structure reveals that the large third extracellular loop (L3) of MFSD6 inserts into the canyon region of the viral capsid, a conserved surface depression near the icosahedral fivefold axis. Key residues within MFSD6 L3 (e.g., Asn200, Leu202, and Glu203) form an extensive network of hydrogen bonds and hydrophobic interactions with residues on viral capsid proteins VP1 and VP3 (20). Alanine substitution of these critical contact residues abrogates binding, confirming the functional importance of the observed interface. This structural insight reveals that despite lacking an immunoglobulin-like fold, MFSD6 engages the canyon similarly to receptors of other picornaviruses, likely initiating conformational changes that lead to uncoating.

## THERAPEUTIC IMPLICATIONS: FROM RECEPTOR DISCOVERY TO DECOY STRATEGIES

### Sialic acid cleavage

DAS181 (Fludase) is a first-in-class recombinant sialidase-human IgG Fc fusion protein that enzymatically cleaves terminal N-acetyl-neuraminic acid residues from airway epithelial glycans, thereby transiently masking the conserved host receptor used by a broad spectrum of respiratory viruses, including influenza A/B, parainfluenza (PIV 1-4), and human metapneumovirus (40–45). By targeting a host determinant rather than a viral protein, DAS181 imposes a high barrier to resistance and retains full activity against oseltamivir-resistant, baloxavir-resistant, and highly pathogenic avian H5/H7 strains (41, 44). The drug is currently in a global Phase III registrational trial for lower respiratory tract PIV infection in immunocompromised patients, having received both Fast-Track and Breakthrough Therapy designations from the U.S. Food and Drug Administration. Rhoden et al. demonstrated that DAS181 treatment potently inhibits infection by sialic acid-dependent EV-D68 strains *in vitro* (46–48). This finding not only confirms the crucial role of sialic acid molecules in mediating EV-D68 infection but also provides a broad-spectrum and convenient therapeutic strategy for the rapid clearance of viral respiratory infections.

### MFSD6-based decoy receptors

The precise characterization of the MFSD6-EV-D68 interface has enabled the rational design of potent antiviral agents based on the “decoy receptor” strategy, revealing a direct path from basic molecular discovery to therapeutic development (49).

Evidence suggests that the role of MFSD6 extends beyond binding to actively promote uncoating. Liu et al. (19) demonstrated that a soluble MFSD6-Fc protein can trigger the release of viral RNA from purified EV-D68 particles in a cell-free assay (19). This finding indicates that the interaction with MFSD6 is sufficient to destabilize the virion and initiate the uncoating process. This dual function, which facilitates both entry and the conformational changes required for genome release, highlights the classification of MFSD6 as a functional uncoating receptor.

To further optimize this approach, Li et al. developed a secreted MFSD6 microbody (secMFSD6 Mb) (49). This agent has a sophisticated dual mechanism of action: it acts as a competitive inhibitor of cellular receptor binding and directly induces irreversible virion destabilization. Treatment with secMFSD6 Mb causes the conversion of intact, full virions to empty capsids, as visualized by electron microscopy, and the release of viral RNA, effectively neutralizing the virus in the extracellular space. This strategy exhibits broad-spectrum activity against diverse EV-D68 strains and shows significant efficacy in human primary airway cells and *in vivo* models.

### Limitations and future directions

Despite the considerable potential of MFSD6-targeted decoys, several critical questions remain unresolved. The physiological role of MFSD6, which is hypothesized to function as a solute transporter, has not been fully elucidated (50), necessitating further investigation into the potential implications of prolonged therapeutic inhibition. Although the high degree of conservation in the MFSD6 binding site implies a substantial genetic barrier to viral resistance, the possibility of viral escape mutants must still be rigorously monitored through empirical studies. Future research should prioritize the optimization of the pharmacokinetic and safety profiles of these biologic agents, including comprehensive assessments of immunogenicity and evaluations of efficacy in more physiologically relevant animal models or against recently circulating EV-D68 clades.

In addition to the aforementioned antiviral strategies, isolated neutralizing monoclonal antibodies have demonstrated potent anti-EV-D68 activity both *in vitro* and *in vivo*. Importantly, these antibodies not only bind to epitopes involved in virus-receptor interactions, thereby preventing viral recognition of host cell receptors, but also induce conformational changes mimicking receptor-mediated uncoating of viral particles, which further enhances their neutralizing capacity. Future research should incorporate advanced structural biology techniques, such as cryo-electron microscopy, to precisely characterize the mechanisms by which vaccine-induced neutralizing antibodies target EV-D68, specifically, their interactions with known attachment factors, including sialic acid, ICAM-5, sGAGs, or MFSD6. These insights will contribute to a deeper understanding of structurally defined immunogens capable of effectively inhibiting viral entry and facilitate the rational design of safer, potent, stable, and broad-spectrum vaccines against enteroviruses.

Moreover, the development of receptor-targeted antiviral strategies could be effectively combined with rapidly advancing research on virus-directed enzyme inhibitors (47, 48, 51–53). Li et al. identified a small-molecule inhibitor, Jun6504, that targets the 2C protein and demonstrates potent antiviral activity against EV-D68 *in vitro* (54). Critically, this inhibitor significantly prevented paralysis in a mouse model of EV-D68-induced neurological disease, providing strong *in vivo* proof-of-concept data for mitigating the most severe clinical outcomes of infection. Future therapeutic approaches may, therefore, rely on the coordinated application of host-directed and virus-directed antiviral agents as a comprehensive strategy to control both the transmission and the pathogenicity of EV-D68.

## THE DYNAMIC MODEL: RECEPTOR SWITCHING AND VIRAL EVOLUTION

The sequential identification of sialic acid, ICAM-5, sGAGs, and MFSD6 as receptors for EV-D68 reveals a fundamental paradigm: viral entry is not a static process but a dynamic interface shaped by evolutionary arms races and tissue-specific constraints. We propose that EV-D68 employs a modular entry system in which glycan attachment factors (e.g., sialic acid, sGAGs) and protein entry receptors (e.g., ICAM-5, MFSD6) can function cooperatively or interchangeably. This plasticity allows the virus to navigate diverse host environments, and its evolution toward protein-receptor dependency may be a key determinant of heightened pathogenicity.

A shift in receptor preference is evident when historical and contemporary strains are compared. Prototype strains, such as Fermon, rely heavily on α2,6-linked sialic acid for both attachment and uncoating. In contrast, many epidemic strains have acquired mutations that enable efficient, sialic acid-independent entry, a transition likely driven by selective pressures associated with adaptation to new ecological niches. This functional plasticity may be facilitated by the topological segregation of receptor-binding sites within the capsid canyon. The recently identified MFSD6 binding site is adjacent to, yet distinct from, the sialic acid pocket, allowing for evolutionary fine-tuning of ligand affinity either individually or cooperatively without compromising essential viral functions.

This type of receptor switching has direct and profound implications for pathogenesis. The pathogenic dichotomy of EV-D68 (i.e., respiratory transmission versus neurological sequelae) may be explained by differential receptor usage. Ubiquitously expressed MFSD6 facilitates robust replication in the respiratory tract, the primary site of infection. In contrast, the ability of certain strains to engage telencephalon-enriched ICAM-5 provides a direct molecular pathway for invading specific brain regions, although the exact physiological significance of this remains to be determined. This model positions receptor preference not merely as a cell-entry mechanism but also as a primary virulence determinant.

From an evolutionary perspective, a broad receptor repertoire provides a robust fitness advantage. It acts as an evolutionary buffer, allowing the virus to maintain infectivity across varying host populations and tissue environments. This flexibility contrasts with that of receptor-monogamous viruses, such as poliovirus, where receptor specificity strictly limits the host range. With respect to EV-D68, the ability to utilize MFSD6, a highly conserved transporter across mammals, may lower the barrier to zoonotic transmission or emergence in new host species.

In conclusion, the evolution of EV-D68 receptor usage illustrates a trajectory toward increased complexity and adaptability. The integration of a ubiquitous entry receptor (MFSD6) with a tissue-specific receptor (ICAM-5) and flexible glycan interactions equips the virus with a formidable capacity for dissemination and pathogenesis. Understanding this dynamic model is crucial: it informs surveillance efforts to track receptor-usage phenotypes in circulating strains and underscores the therapeutic promise of targeting conserved, essential entry interfaces, such as those used by MFSD6, to circumvent viral evasion through receptor switching.

## CONCLUSION

The delineation of the EV-D68 receptor landscape has transitioned from mapping static interactions to deciphering a dynamic system of remarkable plasticity. The identification of MFSD6 as a bona fide broadly expressed entry receptor provides a mechanistic cornerstone for viral tropism and a validated target for therapeutic intervention. However, this discovery also highlights a more complex reality: EV-D68 employs a context-dependent repertoire of receptors (sialic acid, ICAM-5, and MFSD6), whose usage is likely determined by tissue availability, viral strain, and evolutionary selection—moving beyond binary models to a model of adaptive entry.

Defining the hierarchical and spatiotemporal dynamics of receptor engagement *in vivo* is challenging. Although MFSD6 and ICAM-5 are individually sufficient *in vitro*, their relative contributions to respiratory infection versus neuroinvasion in a living host remain unclear. The development of conditional knockout models—ablating MFSD6 specifically in airway epithelia or ICAM-5 in the central nervous system—will be critical for determining whether neuroinvasion is a receptor-directed event or a stochastic consequence of high viremia. Furthermore, the physiological function of MFSD6 remains unclear. Understanding its native role as an orphan transporter is paramount; viral hijacking could induce pathogenesis not only through lytic infection but also by disrupting its endogenous transport function, a possibility that warrants investigation.

The identification of MFSD6 represents a rapid advance; however, it by no means completes the picture of EV-D68 entry. Accumulating evidence points to the potential existence of other host factors that assist EV-D68 entry. The nature of these factors is a primary unsolved question. They could be alternative protein receptors with more restricted expression, novel attachment factors, such as specific glycolipids, or host proteins that facilitate post-binding steps, such as endocytosis or intracellular trafficking. Unbiased screening approaches in MFSD6-deficient backgrounds, coupled with sophisticated biochemical methods, will be essential to deconvolute this complex entry apparatus and determine whether these pathways are strain specific or represent a universal backup system.

Therapeutically, MFSD6-based decoy receptors represent a breakthrough, demonstrating that high-affinity receptor mimetics can achieve potent neutralization. However, the genetic barrier to resistance must be empirically defined. Serial virus passage under decoy selection will reveal whether the conserved canyon-L3 interface is a true vulnerability or if escape via capsid remodeling is feasible. Next-generation decoys could be engineered for increased potency, such as by creating bispecific molecules that simultaneously target the MFSD6 and sialic acid binding sites or by optimizing delivery—for example, by using inhaled mRNA formulations to achieve high local concentrations in the respiratory tract while minimizing systemic immunogenicity.

In the future, proactive evolutionary surveillance is essential. EV-D68 has a documented capacity for receptor switching. Integrating capsid sequencing from clinical isolates with high-throughput phenotyping for receptor dependency will be crucial for anticipating future shifts, such as mutations that refine MFSD6 usage or, more concerningly, enable the engagement of a novel receptor. The goal is to move from reactive characterization to predictive virology.

In summary, the study of EV-D68 receptors is no longer about identifying singular factors but rather about understanding a sophisticated dialog between viral evolution and host cell biology. Interrupting this dialog through targeted therapeutics and vigilant surveillance is the next critical step in mitigating the threat posed by this adaptable pathogen.
